# Global research trends in in-stent neoatherosclerosis: A CiteSpace-based visual analysis

**DOI:** 10.3389/fcvm.2022.1025858

**Published:** 2022-11-08

**Authors:** Heng Wang, Qian Wang, Jie Hu, Ruijing Zhang, Tingting Gao, Shuling Rong, Honglin Dong

**Affiliations:** ^1^Department of Vascular Surgery, The Second Hospital of Shanxi Medical University, Taiyuan, China; ^2^Department of Cardiology, The Second Hospital of Shanxi Medical University, Taiyuan, China; ^3^Department of Nephrology, The Second Hospital of Shanxi Medical University, Taiyuan, China

**Keywords:** in-stent neoatherosclerosis (ISNA/NA), in-stent restenosis (ISR), CiteSpace, visualization analysis, optical coherence tomography (OCT), cardiovascular disease

## Abstract

**Background:**

Recent studies have shown that in-stent neoatherosclerosis (ISNA/NA) is an important cause of late stent failure. A comprehensive understanding of the current state of research in this field will facilitate the analysis of its development trends and hot frontiers. However, no bibliometric correlation has been reported yet. Here, we analyze the relevant literature since the emergence of the concept and provide valuable insights.

**Methods:**

Publications were collected from the Web of Science Core Collection (WoSCC) and PubMed. Microsoft Excel, SPSS and CiteSpace were used to analyze and present the data.

**Results:**

A total of 498 articles were collected, with Japan and Cardiovasc Res Fdn being the main publishing forces in all country/region and institutions. J AM COLL CARDIOL is the journal with the most published and co-cited articles. According to co-citation analysis, optical coherence tomography, thrombosis, implantation, restenosis, drug-eluting stent, and bare metal stent have become more and more popular recently.

**Conclusion:**

ISNA is a niche and emerging field. How to reduce the incidence of ISNA and improve the late patency rate of coronary stents may remain a hot spot for future research. The pathogenesis of ISNA also needs to be explored in more depth.

## Introduction

Cardiovascular disease (CVD) has become the leading cause of death and a major contributor to disability worldwide, and is the leading cause of death in China’s urban and rural populations, accounting for upwards of 40% of disease deaths (1, 2). Percutaneous coronary intervention (PCI), the most common procedure in cardiology (3), is widely used in the treatment of coronary artery disease (4). However, late complications such as in-stent restenosis (ISR) and in-stent thrombosis (IST) have become a serious problem affecting patient prognosis (5). Early studies have found that drug-eluting stents (DES) significantly reduce the incidence of ISR and improve the overall safety and efficacy of PCI compared to bare metal stents (BMS) (6). However, as time lengthens, DES undergoes a “late catch-up phenomenon” in the late stages of stent implantation (5–10 years or more), with no difference in the reduction of ISR and IST rates between the two (7). There is no difference between the two in reducing the incidence of ISR and IST. It has been shown that in-stent neoatherosclerosis (ISNA/NA) is strongly associated with the occurrence of ISR, IST, and other major adverse cardiovascular events (MACE) after stenting (8, 9).

Since 2010 when Kang et al. (10) first proposed ISNA by virtual histological ultrasound assessment of the tissue characteristics of neoplastic endothelium after stent implantation. Since Otsuka et al. (11) studied ISNA using postmortem and intravital imaging methods, the understanding of ISNA has been greatly improved. ISNA is defined as the transformation of the normal intimal layer within the stent into atherogenic neointima, a process mediated by the accumulation of foamy macrophages with or without calcification (12). The incidence of ISNA increases with time to stent placement (13). Early ISNA often forms clusters of foamy macrophages around the stent, which accumulate and apoptosis in the neointima to form fibrous plaques and necrotic cores containing large amounts of free cholesterol and non-cellular debris. As dead foamy macrophages continue to accumulate, the necrotic core expands further, forming thin-cap fibroatheromas (TCFA), which eventually lead to rupture of the plaque within the stent (14, 15). Currently, the mechanism of ISNA development and rapid progression remains unclear, and whether there is a link with natural atherosclerosis remains controversial.

Bibliometrics is an effective method for describing trends in research fields and is widely used in medical research (16). CiteSpace is a tool for visualizing and analyzing new trends and developments in science, helping researchers to quickly identify existing research progress, discover and track research hotspots (17). In recent years, there are many studies on ISNA, but there is no bibliometric analysis yet. In this paper, we use bibliometric analysis to qualitatively and quantitatively evaluate the scientific progress of ISNA, with a view to identifying new trends and hotspots in the field and predicting future research priorities.

## Methods

### Search strategies

The Web of Science database was used as the original source of the literature data, supplemented by the PubMed database. The authors searched WoS in July 2021 and created a search formula with the topic “in-stent neoatherosclerosis”: TS = (”neoatherosclerosis” OR “in-stent neoatherosclerosis”). The study inclusion criteria were: database: Web of Science Core Collection; language: English; document type: All document types; search period: all years (1985–2021); and search deadline of July 1, 2021. The final search yielded 498 documents: exported in “Other file format,” with the record content “Full record with cited references,” in “Plain text” format for Exported to EndNote Desktop with the following records: author, title, source publication, and abstract for EndNote software check.

### Data collection and analysis

Using a search strategy to identify and download the complete WoS records relevant to this study, two researchers independently extracted data for each document, including author name, nationality, institution, article title, year of publication, journal name, grant, keywords, and abstract. The two researchers then independently analyzed the extracted data using IBM SPSS Statistics 26. Suitable for inclusion in this study were studies related to ISNA, including clinical cases, mechanistic studies, molecular biology analyses, drug-coated stent-related studies, and review articles. Studies were excluded from this analysis if they were not relevant, or if the corresponding full text was not available.

Using CiteSpace 5.7.R2 software for data visualization and analysis, firstly, create four folders “input” “output” “project” “data” and put the filtered text data named “download_XX” into “input” folder. “data” four folders, the filtered text data named “download_XX,” into the “input” folder, after data conversion by CiteSpace software, output to “output” folder, and copy the output file to “data” folder. Create a self-named “New Project,” “Project Home” for the “project” folder,” “Data Directory” for the “data” folder. “Data Directory” for the “data” folder, the rest of the settings for the default, run CiteSpace software for data analysis.

The quality of literature publication by country/region and author is assessed based on metrics including total citations, average citations, IF index, etc. These metrics are used to visualize data using software such as SPSS, CiteSpace, etc. CiteSpace software is used to construct visual bibliometrics based on collaborative networks, co-occurrence analysis, and co-citation analysis. The information used includes author names, institutions, nationalities, journals, keywords, citations, etc. The co-occurrence networks are constructed to visualize the important scientific terms in these literatures.

## Results

### Global publication characteristics

#### Articles listed by country/region

**FIGURE 1:**
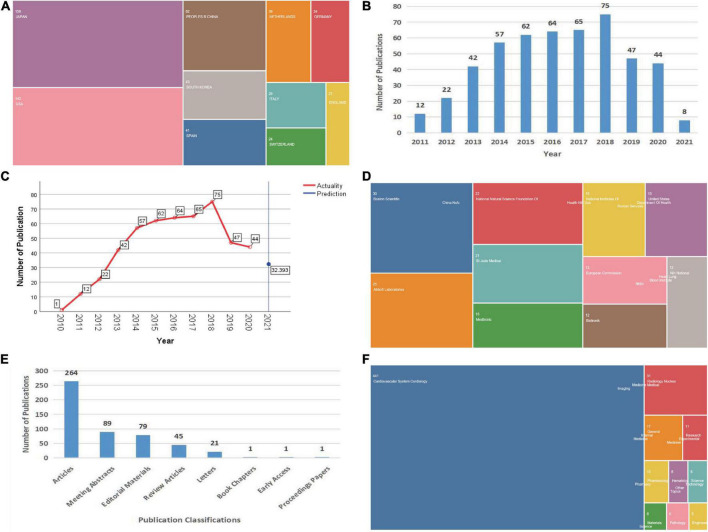
Global characteristics of articles published in ISNA. **(A)** Country/region of origin of articles. **(B)** Annual publication volume of articles. **(C)** Future publication forecast curve of articles. **(D)** Source of project fund support. **(E)** Category classification of articles. **(F)** WoS catalog classification of research areas.

A total of 498 articles that met our search criteria were retrieved from WoS. The global contributions in the field of ISNA research were arranged by color (Figure 1A), indicating that Japan contributed the most articles in this field (158; 31.73%), followed by the United States (142; 28.51%), China (62; 12.45%), South Korea (43; 8.63%), and Spain (41; 8.23%). A total of 351 documents were retrieved by PubMed search, and the aggregated WoS data were used in CiteSpace software for visual analysis.

#### Articles by year

Publications in this field are listed by year in the figure (Figure 1B). The highest number of articles published was in 2018 (75; 15.06%), and the overall trajectory from 2011 to 2018 shows a steady growth trend in the literature related to ISNA research. The number of articles published has decreased slightly in the last 3 years, but still maintains a steady output of articles.

#### Global publishing trends forecast

The time series conventional model of SPSS software was used to forecast the future publication trends in this field (Figure 1C). Only 32 articles in 2021, which shows a sharp decline in the number of articles published in this field since 2019 and still a downward trend in the future.

#### Trends in fund support

Donor support in this area is listed in the figure (Figure 1D), with Boston Scientific providing the largest amount of grant program support in this research area (30; 6.02%), followed by Abbott (25; 5.02%), the National Natural Science Foundation of China (22; 4.42%), St. Yoda Medical (21; 4.217%), and Medtronic (16; 3.21%), among others.

#### Literature categories

Dividing all articles by category (Figure 1E), the highest percentage of these 498 articles (264 articles; 53.01%) was found in the category of thesis, followed by conference abstracts (89 articles; 17.87%), editorial materials (79 articles; 15.86%), review articles (45 articles; 9.04%), and letters (21 articles; 4.22%).

#### Research areas

In the WoS catalog, a total of 25 research areas are shown (Figure 1F). Most of the literature is focused on “cardiovascular diseases” (441; 88.55%), “radiological nuclear imaging” (31; 6.23%), “general internal medicine” (17; 3.41%) and “research laboratory medicine” (11; 2.21%), indicating that ISNA is a multifaceted and multidisciplinary field that covers a wide range of societal benefits. In addition, the latest research themes are “psychiatry,” “physics,” “oncology,” and “genetic genetics,” all of which will appear for the first time in 2019.

#### Publication of magazines

As the table (Table 1) lists the top 10 journals with the highest number of publications, the number of articles published is above 15. Among them, JCR Q1 4, Q2 2, Q3 4, the overall quality of publications is high.

**TABLE 1:** The top 10 journals with the largest number of articles.

Rank	Journal	Count	IF(2021)	JCR(2021)
1	J AM COLL CARDIOL	59	27.203	Q1
2	EUR HEART J	42	35.855	Q1
3	JACC-CARDIOVASC INTE	31	11.075	Q1
4	CIRCULATION	24	39.918	Q1
5	INT J CARDIOL	24	4.039	Q3
6	EUROINTERVENTION	23	7.728	Q2
7	CATHETER CARDIO INTE	20	2.585	Q3
8	CIRC-CARDIOVASC INTE	18	7.514	Q2
9	J CARDIOL	18	2.974	Q3
10	CIRC J	16	3.35	Q3

### Cooperation network analysis

**FIGURE 2:**
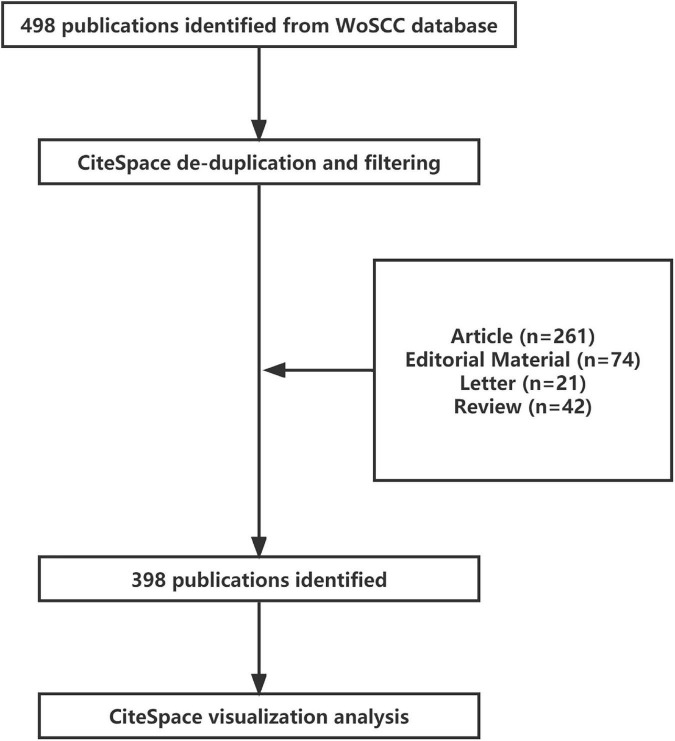
Flowchart of the screening process.

A total of 498 articles were searched through WoS from January 1, 2011 to July 1, 2021, and after importing them into CiteSpace 5.7.R2 software, the data were de-duplicated and cleaned, and zero duplicates were found, and a total of 398 articles were extracted for analysis (Figure 2). These 398 articles were all in English language, from 267 research institutions in 62 countries/regions, by 392 authors, and published in 109 journals.

#### Country/Region

**FIGURE 3:**
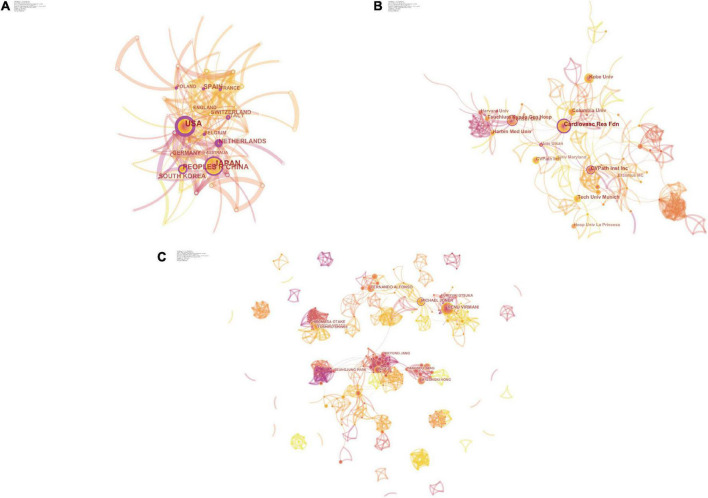
The collaboration analysis of countries/regions, institutions, and scholars in the field of ISNA. The size of the nodes represents the number of posts; the connection between the nodes represents the cooperation; the thickness of the connection represents the strength of the cooperation. Betweenness centrality is a measure of the importance of nodes in the network (in addition to degree centrality, proximity centrality, etc.), and such nodes play the role of “bridges” in the network and are highlighted with purple circles. **(A)** The collaboration network of countries/regions. **(B)** The collaboration network of institutions. **(C)** The collaboration network of scholars.

By visualizing the country/region collaboration network from 2010 to 2021 through CiteSpace software and highlighting the mapping (Figure 3A and Supplementary Figure 1A), the countries shown published at least 10 articles. The top 10 countries/regions with the highest number of papers (Supplementary Table 1) accounted for 75.77% of the article output, with China being the only developing country. Japan is the country with the most published articles, with 146, maintaining a partnership with 10 other countries, followed by the United States (136), China (61), South Korea (42), and Spain (41). It is noteworthy that Japan experienced a sudden increase in scientific output in 2013, and similar phenomena were observed in the United States from 2010 to 2011, Korea from 2010 to 2012, and China from 2019 to 2021. This sudden increase in scientific output, also known as a “Burst” may be the result of a breakthrough in the field. The intensity of the burst is strongest in China, at 5.1, much higher than in other countries.

Higher centrality values, also known as mediator centrality, represent the more active and close role a node plays in cooperative relationships with other nodes, and individuals with high centrality are marked with purple circles in the graph. USA (0.29), NETHERLANDS (0.23), SPAIN (0.19), ITALY (0.18), and POLAND (0.16) have high intermediary centrality (Figure 3B) and play an important role as a bridge in the network of national cooperation in this field. China and Japan don’t have high centrality, both at 0.13, although they have a higher number of publications.

#### Institutions

All 398 articles were published at 267 research institutions (Figure 3B and Supplementary Table 2), and the institutions shown had at least 8 article outputs. Among them, the top 10 high-output institutions, accounting for about 25.5% of the total output of research institutions, are seen in the research institution collaboration network Cardiovasc Res Fdn, Yonsei Univ, CVPath Inst Inc, Kobe Univ, Tech Univ Munich, and many other research institutions form the major research teams with the highest number of publications. The number of articles published is at the top. Among them, Cardiovasc Res Fdn is the most active institution, publishing 30 articles and collaborating with 20 institutions. Based on the highly cited articles of this institution, we found that its research themes mainly lie in the pathological characterization and advantages of optical coherence tomography (OCT) for ISNA (18, 19) and the prognosis of different types of stent implantation (20, 21).

In addition, Harvard Univ saw an article publication burst from 2012 to 2015, ranking first with an intensity of 4.77. Kurashiki Cent Hosp (2016–2017), Univ Bern (2014–2016), and CVPath Inst Inc (2011–2014) all saw publication bursts. Cardiovasc Res Fdn centrality 0.27 ranked first, followed by Univ Amsterdam (0.18) and Amer Heart Poland (0.15) (Supplementary Figure 1B).

#### Authors

There are 392 authors for all 398 articles, which shows the concentration of researchers in this field (Figure 3C). In the author collaboration network, each node represents an author, and the size and color of the author’s name is proportional to the number of articles published; the line between nodes represents the collaboration between two people, and the thicker the line, the stronger the collaboration; the color of the line represents the year of collaboration.

The authors with the highest number of publications are considered as the core researchers in this research area, with RENU VIRMANI (30) ranking first, followed by MICHAEL JONER (21), FERNANDO ALFONSO (19), BO YU (18), and KENICHI HIRATA (16), forming an aggregation centered on themselves A large group of researchers. Among them, Renu Virmani and Michael Joner have always maintained their research enthusiasm and their literature output is stable; while Fernando Alfonso, Bo Yu, Kenichi Hirata and others, who had a lot of early research work, have decreased the number of publications in the last five years; Kenichi Hirata, Hiromasa Otake, and Toshiro Shinke have collaborated extremely closely and published more articles, but have not formed collaborations with other researchers.

RENU VIRMANI, as the most active researcher in this field, has focused on the comparison of different types of drug-eluting stents (21), and the adverse outcomes of ISNA (5). MICHAEL JONER has the highest centrality of 0.12 and has a high level of communication and collaboration with other researchers (Table 2). In addition, there are many newcomers to the field, and the volume of publications, although not outstanding, plays an important role in enriching the collaborative grid as well as promoting the latest research. Overall, it seems that, except for the teams that have formed a certain degree of aggregation, many scholars and teams lack collaboration with each other, and some important teams with a large number of early publications have even withdrawn from the field gradually.

**TABLE 2:** The top 10 authors with the highest number of articles and their representative articles.

Rank	Author	Count	Centrality	Total citations	Selected publications
1	RENU VIRMANI	30	0.05	2174	The Pathology of Neoatherosclerosis in Human Coronary Implants Bare-Metal and Drug-Eluting Stents (20)
2	MICHAEL JONER	21	0.12	957	Stent thrombosis and restenosis: what have we learned and where are we going? The Andreas Gruntzig Lecture ESC 2014 (22)
3	FERNANDO ALFONSO	19	0.05	549	Current Treatment of In-Stent Restenosis (23)
4	BO YU	18	0.01	250	Predictors for Neoatherosclerosis A Retrospective Observational Study From the Optical Coherence Tomography Registry (24)
5	KENICHI HIRATA	16	0	112	The impact of in-stent neoatherosclerosis on long-term clinical outcomes: an observational study from the Kobe University Hospital optical coherence tomography registry (25)
6	TOSHIRO SHINKE	15	0	112	The same as 5
7	HIROMASA OTAKE	15	0	112	The same as 5
8	MYEONGKI HONG	14	0	216	Optical Coherence Tomographic Observation of In-Stent Neoatherosclerosis in Lesions With More Than 50% Neointimal Area Stenosis After Second- Generation Drug-Eluting Stent Implantation (26)
9	YANGSOO JANG	14	0.01	336	The same as 4
10	IKKYUNG JANG	14	0	293	The same as 4
10	FUMIYUKI OTSUKA	14	0.01	1525	The same as 1
10	SEUNGJUNG PARK	14	0.02	759	In-Stent Neoatherosclerosis A Final Common Pathway of Late Stent Failure (5)

### Keyword co-word analysis

#### High-frequency keywords

**FIGURE 4:**
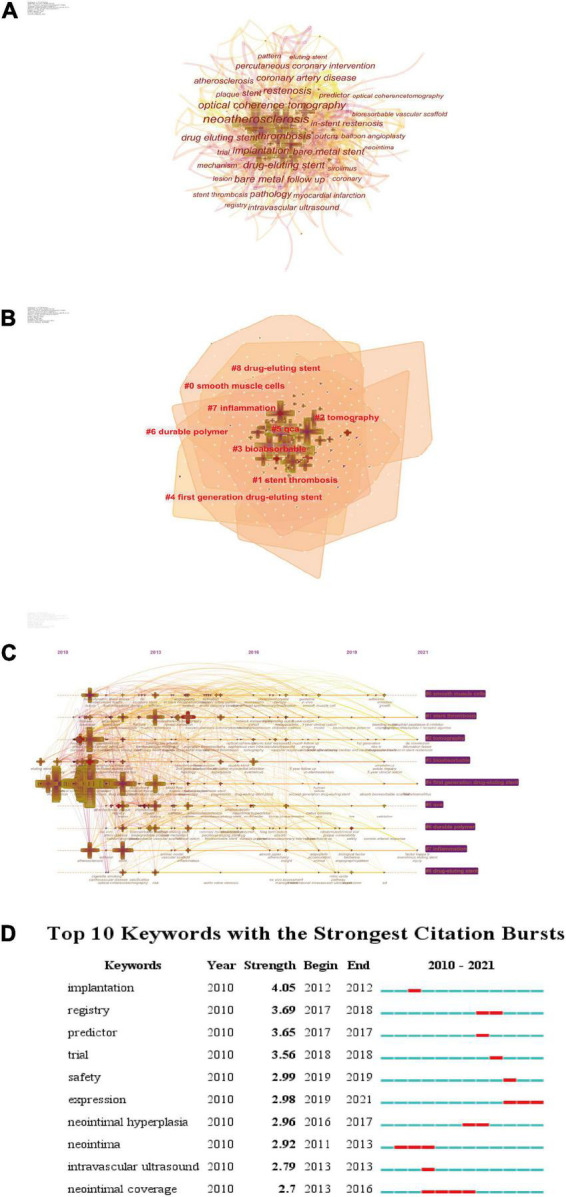
The keyword analysis network. Burst detection: used to detect sudden changes in subject, literature, author, and journal citation information. **(A)** The network of co-occurring keywords. **(B)** The network of co-occurring keywords clusters based on LLR algorithm. **(C)** Timelines of co-citation clusters. **(D)** The top 50 keywords with the strongest citation bursts.

The keyword co-occurrence profile (Figure 4A) was obtained from the keyword analysis of the literature by CiteSpace software. The module value (Modularity) *Q* = 0.4423, the module value of 0.4–0.8 usually forms a small number of natural network clusters, which meets the analysis requirements; the average contour value (Weighted Mean Silhouette) *S* = 0.7579, when *S* ≥ 0.5, it is generally considered that the cluster is more reasonable and meets the analysis requirements. Each node represents a keyword, the size of the “ten” graph represents the frequency of the keyword, the linkage of two nodes represents the co-occurrence of two keywords, the color of the linkage represents the time, the thickness represents the degree.

According to Donohue et al. (27) proposed formula for dividing high-frequency and low-frequency keywords, the threshold T for high-frequency keywords in this study was calculated as (I1 is the total number of keywords with word frequency of 1). T≈17.41 was obtained by substituting the formula, so keywords with word frequency ≥ 18 were extracted as high-frequency keywords, and 34 high-frequency keywords were obtained, which can show the whole picture of ISNA research.

The high frequency keywords were organized (Table 3). Among them, neoatherosclerosis appeared 247 times, which was far ahead, followed by optical coherence tomography (140), thrombosis (120), implantation (114), and restenosis (106). It can be seen that ISNA research areas focus on optical coherence tomography, thrombosis, grafting, revascularization, drug-eluting stents, and bare stents. In addition, coronary artery disease had the highest centrality (0.13), angioplasty (0.12), acute myocardial infarction (0.12), and atherosclerosis (0.11) had the highest centrality. It plays an important mediating role in enriching the co-occurrence map network.

**TABLE 3:** High frequency keywords list.

Keywords	Frequency	Keywords	Frequency	Keywords	Frequency
Neoatherosclerosis	247	Stent	54	Stent thrombosis	25
Optical coherence Tomography	140	In-stent restenosis	47	Pattern	22
Thrombosis	120	Atherosclerosis	46	Coronary	21
Implantation	114	Percutaneous coronary intervention	43	Sirolimus	21
Restenosis	106	Outcome	39	Balloon angioplasty	21
Drug-eluting stent	105	Intravascular ultrasound	38	Bioresorbable vascular scaffold	20
Bare metal	103	Myocardial infarction	33	Optical coherence tomography	19
Bare metal stent	78	Mechanism	32	Eluting stent	19
Drug eluting stent	76	Trial	31	Registry	19
Coronary artery disease	64	Plaque	27	Neointima	18
Pathology	60	Predictor	27		
Follow up	55	Lesion	25		

#### Keyword clustering analysis

The keyword clustering mapping was obtained by clustering the keywords of the literature through CiteSpace software (Figure 4B and Table 4). The layout was obtained by taking the year of citation publication as the *X*-axis and the cluster number as the *Y*-axis. As seen in the figure, the keywords are clustered into 9 labels, and the clustering labels are derived from the LSI/LLR/MI algorithm respectively, and the cluster number is inversely proportional to the cluster size. These clustered keywords can reflect the research hotspots in the field of ISNA, which is significant for predicting the development pattern and new directions of research in this discipline. The clusters were organized to obtain the table.

**TABLE 4:** Details of clusters.

Cluster ID	Size	Mean (Year)	Top terms (LSI)	Top terms (LLR, p-level)	Top terms (MI)
#0	50	2015	Neoatherosclerosis	Smooth muscle cells (4.05, 0.05)	Cholesterol (0.51)
#1	44	2015	Stent thrombosis	Stent thrombosis (11.9, 0.001)	Plaque angiogenesis (0.4)
#2	40	2015	Stent thrombosis	Tomography (8.6, 0.005)	Coronary vessels (0.36)
#3	40	2013	Coronary artery disease	Bioabsorbable (4.67, 0.05)	Bioabsorbable (0.28)
#4	39	2013	Drug-eluting stent	First generation drug-eluting stent (3.5, 0.1)	First generation drug-eluting stent (0.6)
#5	38	2014	Optical coherence tomography	qca (9.02, 0.005)	Peroneal artery (0.32)
#6	35	2016	Percutaneous coronary intervention	Durable polymer (5.5, 0.05)	Absorb (0.31)
#7	21	2017	Drug-eluting stents	Inflammation (11.37, 0.001)	Rabbits (0.15)
#8	21	2017	Atherosclerosis	Drug-eluting stent (des) (5.81, 0.05)	Drug-eluting stent (des) (0.13)

As seen from the LLR algorithm (Supplementary Table 3), clusters #0 and #7# are mainly studies related to the potential pathogenesis of ISNA; clusters #1 are mainly studies related to the mechanism of late stent failure and complications; clusters #2 and #5 are mainly studies related to the clinical imaging diagnosis of ISNA; clusters #3, #4, #6, and #8 focus on the mechanism related to the type of stent and the occurrence of ISNA studies.

#### Keyword timeline analysis

The keyword time line analysis of the literature was obtained by CiteSpace software to obtain the keyword time line mapping (Figure 4C). The analysis shows that in the first 5 years of ISN research, the research direction is relatively concentrated, mainly around several thematic keywords, such as optical coherence tomography, thrombosis, restenosis, drug-eluting stent, bare metal, etc.; as time progresses, the research direction As time progressed, the research direction gradually broadened and a series of emerging keywords emerged, such as everolimus, neointimal hyperplasia, proliferation, tissue characteristics, etc. It can be found that the research on ISN is going deeper from clinical diagnosis to pathogenesis and therapeutic means, and is being explored in multiple directions, from a wide perspective and at a deeper level.

In addition, the time line diagram can also show the time span and research progress, in which cluster #4 has the most literature, and its research is important throughout this cluster area. The keywords of clusters #1, #2, #3, and #5 have purple circles in the shape of a cross, indicating a high degree of centrality, which is the hub between the connections of different clusters.

#### Keyword bursts

The keyword bursts of the literature were analyzed by CiteSpace software to obtain the keyword burst profile (Figure 4D). The analysis shows that keywords such as neointimal coverage, expression, and neointima have been highlighted for a long time, indicating a high research fervor in the study of mechanisms such as neonatal endosomes. Among them, replantation has the highest burst intensity of 4.05, while registry (3.69), predictor (3.65) and trail (3.56) have higher burst intensity, indicating that these keywords were the hot research topics at that time in the corresponding time period. Keywords such as expression, safety, and trial have been highlighted in the past 3 years, indicating that research hotspots focus on pathogenesis, risk assessment, and clinical trials.

### Co-citation analysis

#### Literature co-citations

**FIGURE 5:**
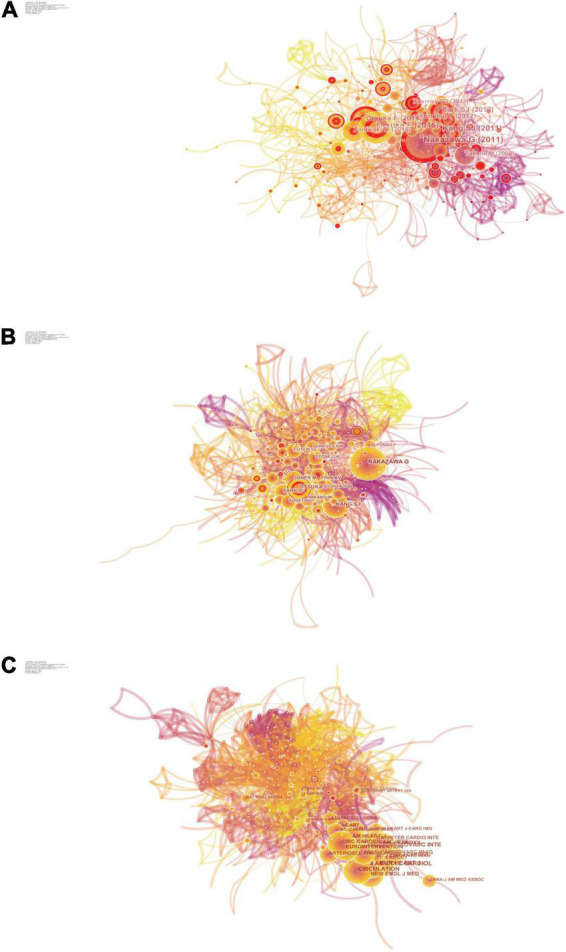
Co-citation map. **(A)** The network of co-cited references. **(B)** The network of co-cited authors. **(C)** The network of co-cited journals.

The top 10 articles with the highest frequency of co-citation in the field of ISNA research were compiled and listed by CiteSpace software to obtain the co-citation and highlighting maps of the literature (Figure 5A, Supplementary Figure 2A, and Table 5). In 2011, Nakazawa et al. (20) first explained the pathological mechanism of ISNA and proposed a better definition of ISNA, which was considered as a cluster of lipid-filled foamy macrophages accumulating in the neoplastic endothelium with or without necrotic core formation. It was also reported for the first time that ISNA occurs more frequently and appears earlier in DES compared to BMS. Kang et al. (18), by performing OCT imaging in patients after DES implantation, found that patients with failed stent implantation were often associated with ISR (either stable or unstable) and found neointimal hyperplasia, neointimal rupture and thrombosis within the stent. Suggesting that ISNA may be an important mechanism of DES failure, especially late after stenting. Park et al. (5) found evidence that ISNA is an important substrate for ISR and LST, especially in the late stages of stent placement. Given the rapid progression within DES, early detection of ISNA may help improve the long-term prognosis of patients with DES implantation. Otsuka et al. (21) found that the second-generation cobalt-chromium everolimus-eluting stent CoCr-EES showed greater stent coverage, less inflammation, less fibrin deposition, and less LST and VLST compared to sirolimus-eluting stents (SES) and paclitaxel drug-eluting stents (PES) at autopsy. These studies play an important role in understanding the clinical features and pathological mechanisms of ISNA.

**TABLE 5:** The top 10 co-cited references sorted by the number of citations.

Rank	References	Citation count	Journal	IF (2021)	Author	Year	Centrality
1	The pathology of neoatherosclerosis in human coronary implants bare-metal and drug-eluting stents (20)	134	J AM COLL CARDIOL	24.094	Nakazawa G	2011	0.01
2	Optical Coherence Tomographic Analysis of In-Stent Neoatherosclerosis After Drug-Eluting Stent Implantation (18)	97	CIRCULATION	29.69	Kang SJ	2011	0.02
3	The coronary substrate determines prognosis in acute coronary syndromes: the kaleidoscope has been shaken again! (28)	93	EUR HEART J	29.983	Otsuka F	2015	0.01
4	In-Stent Neoatherosclerosis: A Final Common Pathway of Late Stent Failure (5)	65	J AM COLL CARDIOL	24.094	Park SJ	2012	0.01
5	Pathology of Second-Generation Everolimus-Eluting Stents Versus First-Generation Sirolimus- and Paclitaxel-Eluting Stents in Humans (21)	60	CIRCULATION	29.69	Otsuka F	2014	0.04
6	Predictors for neoatherosclerosis: A Retrospective Observational Study From the Optical Coherence Tomography Registry (24)	50	CIRC-CARDIOVASC IMAG	7.792	Yonetsu T	2012	0.02
7	Mechanisms of Very Late Drug-Eluting Stent Thrombosis Assessed by Optical Coherence Tomography (29)	38	CIRCULATION	29.69	Taniwaki M	2016	0.06
8	Appearance of Lipid-Laden Intima and Neovascularization After Implantation of Bare-Metal Stents: Extended Late-Phase Observation by Intracoronary Optical Coherence Tomography (30)	33	J AM COLL CARDIOL	24.094	Takano M	2009	0.01
9	Consensus Standards for Acquisition, Measurement, and Reporting of Intravascular Optical Coherence Tomography Studies: A Report From the International Working Group for Intravascular Optical Coherence Tomography Standardization and Validation (31)	28	J AM COLL CARDIOL	24.094	Tearney GJ	2012	0.03
10	Optical Coherence Tomography Findings in Patients With Coronary Stent Thrombosis (9)	27	CIRCULATION	29.69	Adriaenssens T	2017	0.07

In addition, Kang et al. (18) and Nakazawa et al. (20), both of which have burst intensities higher than 10 and lasted for more than 3 years, are two highly cited articles that have a wide impact in the ISNA field and lay the foundation for research in the ISNA field.

#### Authors’ co-citations

Author co-citation analysis is a bibliometric method to analyze the literature published by different authors that are simultaneously cited by other literature, and thus to study the thematic relationships between authors. In the author co-citation and highlighting mapping (Figure 5B, Table 6, and Supplementary Figure 2B), Nakazawa, Kang, and Otsuka are both highly cited authors and their publications are highly cited literature, and they have had a profound impact on the entire research field of ISNA, although they have not published many articles. In addition, FUJII K, BYRNE RA, and CHEN MS all have centrality greater than 0.1, and nodes with high centrality are usually considered to be turning points or critical points in a field (32), indicating that they have published articles of great significance in the field of ISNA.

**TABLE 6:** The top 10 co-cited author sorted by the number of citations.

Rank	Cited author	Count	Centrality	Year
1	NAKAZAWA G	275	0.01	2010
2	KANG SJ	169	0.01	2011
3	OTSUKA F	156	0.02	2014
4	JONER M	115	0.06	2011
5	FINN AV	97	0.01	2011
6	PARK SJ	96	0.01	2013
7	YONETSU T	90	0.02	2013
8	GONZALO N	89	0.03	2011
9	TAKANO M	84	0.03	2011
10	STONE GW	76	0.02	2011

#### Journal co-citations

JACC and CIRCULATION have the highest co-citation frequencies (Figure 5C, Table 7, and Supplementary Figure 2C) and are the core journals in the field and an important source for understanding the progress and frontier hotspots of ISNA research. CIRC RES has the highest centrality and has some influence in the field.

**TABLE 7:** The top 10 co-cited journal sorted by the number of citations.

Rank	Count	Cited journal	Centrality	Cited journal
1	359	J AM COLL CARDIOL	0.14	CIRC RES
2	340	CIRCULATION	0.11	CARDIOVASC RES
3	256	JACC-CARDIOVASC INTE	0.11	AM J PHYSIOL-HEART C
4	250	EUR HEART J	0.11	ANN BIOMED ENG
5	233	CIRC-CARDIOVASC INTE	0.09	BIOMATERIALS
6	210	EUROINTERVENTION	0.08	ANN INTERN MED
7	200	NEW ENGL J MED	0.07	NAT REV CARDIOL
8	184	AM J CARDIOL	0.07	J CARDIOL
9	168	LANCET	0.07	BMJ-BRIT MED J
10	165	JACC-CARDIOVASC IMAG	0.07	NATURE
10			0.07	CELL TISSUE RES

## Discussion

### In-stent neoatherosclerosis global research quality

Based on our selection criteria in the WoS database from January 1, 2011 to July 1, 2021, a total of 392 authors from 267 research institutions in 62 countries/regions with 398 ISNA-related research publications in 109 journals were extracted after software data de-duplication and cleaning. By conducting a bibliometric and visual analysis, we found that the number of publications has been increasing between 2010 and 2018. However, the number of publications per year in the last 3 years is in a relatively stable state, indicating that the field of ISNA is still a relatively popular research direction.

The analysis of the distribution of countries/regions and institutions helps to promote teamwork and global collaboration in a given field. In this study, we can see that the country with the highest number of publications is the United States (136), followed by China (61) and Korea (42). Moreover, the United States has the highest centrality of 0.29, indicating that it plays an important role as a bridge in inter-country cooperation. In addition, the institution with the most published papers was Cardiovasc Res Fdn (30), followed by Yonsei Univ (21) and CVPath Inst Inc (19). It shows that researchers from the United States, China and Korea are the core research force in this field, and these two countries are the most influential in the field of ISNA, and this distribution may be related to the economic development and investment in academic research in these countries. In addition, according to Figure 2 it can be seen that there was close cooperation among core research countries, such as the United States, China, and South Korea before 2015, after which some non-core countries started to increase their cooperation. And among the institutional cooperation networks, core research institutions started to work closely together in 2018 and after, after the lack of cooperation before. In view of this, strengthening communication and cooperation among national institutions has far-reaching implications for promoting further development in this field.

The analysis of journals and co-cited journals can help researchers to choose the right journal to submit their papers. Among the top ten journals in terms of number of publications, JCR Q2 and above accounted for six, and the highest IF was J AM COLL CARDIOL (IF = 27.203). The journals with the highest co-citation frequency were JACC (359) and CIRCULATION (340) with the highest co-citation frequency, and the JCR Q1 division accounted for eight of the top ten journals, with the highest IF being LANCET (IF = 202.731). These studies suggest that ISNA research has great clinical value and is favored by many high-quality, high-impact journals.

The analysis of author collaboration network and author co-citation helps to analyze the research direction of the authors and provide further guidelines. RENU VIRMANI published the highest number of articles (30), followed by MICHAEL JONER (21), FERNANDO ALFONSO (19); NAKAZAWA G was the most co-cited authors (275), followed by KANG SJ (169), OTSUKA F (156), indicating their potentially outstanding contributions in the field.

### Current status of in-stent neoatherosclerosis research and future prospects

In-stent restenosis and ISNA as well as late stent thrombosis are considered to be the main causes of long-term PCI failure (33). Solitary and early (<1 year) ISR is characterized by the proliferation and migration of vascular smooth muscle cells (VSMCs), leading to the development of significant intimal hyperplasia (34). In contrast, the formation of ISNA has become a key factor predisposing to long-term coronary complications, including late ISR (>1 year) and very late stent thrombosis (20). Despite the use of next-generation drug-eluting stents, late stent failure rate has not been effectively controlled, and ISNA is a factor that cannot be ignored (35). Therefore, it is extremely necessary to pay attention to the research progress in the field of ISNA, which may provide new methods for ISR prevention and treatment.

In-stent neoatherosclerosis is defined as the transition from a normal intimal layer to an atherogenic neoplastic intima within the stent, a process mediated by the accumulation of lipid macrophages with or without calcification (12). Whereas native coronary atherosclerosis takes considerable time (decades) to develop, ISNA forms within months to years after stent placement, suggesting that these are two very different pathological mechanisms (36). The early pathology of ISNA is characterized by the accumulation of lipid-laden foamy macrophages in the neointima after stenting, and is not associated with native atherosclerosis (20). Subsequently, the accumulation of foam macrophages in the neointima or luminal surface can induce the formation of thin cap fibrous atherosclerosis (TCFA), leading to complications such as in-stent plaque rupture and thrombosis (37). In addition, calcification can occur within the neoplastic intima, especially in stents that have been implanted for a long time. Calcification can occur in both BMS and DES, and paclitaxel DES may be associated with persistent fibrin deposition within the stent (38). In addition, different stent types affect the formation of ISNA. Compared to BMS, DES showed earlier ISNA and plaque rupture (20, 26, 39). Bioresorbable stents (BVS) have a much lower incidence of ISNA and late stent failure rate than DES (40, 41). BVS has been used to treat in-stent thrombosis caused by ISNA plaque rupture (42). The mechanism of ISNA formation is still unknown. The mechanism of ISNA formation is still unclear, and incomplete coverage and delayed repair of endothelial cells are important causes. This inability to maintain a fully functional endothelium, the irregular size of these cells, which do not follow the direction of blood flow, the formation of poor cellular junctions between cells, the reduced expression of antithrombotic molecules, and the reduced production of nitric oxide (36, 43). Endothelial insufficiency leads to lipid penetration, which is the initiation stage of ISNA formation. In addition, chronic inflammation caused by macrophage migration, formation of foam cells and other inflammatory response plays an important role in the progression of ISNA (20, 44–46).

The clinical diagnosis of ISNA currently relies on vascular imaging techniques. Optical coherence tomography (OCT) is an imaging technique used to assess the microstructure of coronary arteries, which allows a more detailed description of the superficial structure of the vessel wall compared to intravascular ultrasound (IVUS) (47, 48). Especially in the field of ISNA, it is considered to be the gold standard for diagnosis (49–51). In addition, several new imaging techniques are emerging in the field of intravascular imaging and atherosclerosis research. Sunwon et al. (52) first demonstrated a simultaneous structural and biochemical assessment of high-risk plaques in the beating swine coronary arteries using a fully integrated OCT-FLIm and a 2.9-F low-profile dual-modal catheter. Notably, NIR-II/IIa/IIb *in vivo* imaging (53), magnetic nanoparticle targeting (54), two-photon microscopic imaging, micro-MRI, micro-CT and photoacoustic microscopy techniques can obtain higher resolution *in vivo* imaging, making it possible to visualize the microvascular system and image subcellular structures (55–58).

Current studies of ISNA rely on human imaging observations and histopathological analysis, but there are also some more commonly used animal models. In experimental animal models of coronary intervention, most of the information on coronary ISR and DES after implantation is derived from the porcine model (59–62). Using Porcine model, Suna et al. (59) demonstrated that aggrecan and aggrecanases play vital role in the vascular injury response after stent implantation. However, because the stent implantation time was too short, it does not indicate whether it is related to the formation of ISNA. New Zealand White (NZW) rabbits are widely used as an animal model for the study of atherosclerosis. In rabbits, balloon dilation and stenting of major arteries such as the common carotid artery, abdominal aorta, and iliac artery can be used to study the mechanism of injury in ISNA research (63–66). In addition, implantation of coronary stents in the abdominal aorta of apoE–/– rats is a good model for studying ISR after stent placement and can potentially be used to investigate the mechanisms of injury associated with ISNA (67). These animal models play a great positive role in exploring the mechanism of ISNA formation.

Current studies of ISNA are mostly seen in imaging and histopathological analysis of patients, and animal models also play an important role in the study of ISNA injury mechanisms. Case reports, retrospective studies, and prospective studies dominate, and clinical studies are currently the trend and relatively lacking. In the author’s opinion, current studies should target stent placement patients for comprehensive follow-up and strive to obtain valid imaging and pathological data. A multicenter collaborative approach should be adopted to increase the sample size of patients to obtain more realistic and reliable results. And animal experiments and *in vitro* experiments should also be given attention and are important to study the mechanism of ISNA injury. In addition, multi-omics analysis of human tissues or single-cell sequencing based on ethical requirements is expected to obtain more information at the genetic level.

### Limitations

In this study, we only performed visual analysis of the Web of Science database with the CiteSpace software; therefore, this strategy may miss papers published in other databases. In addition, the search strategy was set up to obtain the most comprehensive data possible, which does not guarantee that all included articles are completely relevant to the ISNA research topic. It is known that older literature has a higher chance of being cited and confirmed, which could potentially lead to a time lag in which recent groundbreaking research results have not yet received sufficient attention. In addition, the software is not able to capture all authors information and distinguish the order of authors for the time being, which may lead to less accurate evaluation of the contributions of researchers in the field. Therefore, we need to look at the advantages and limitations of this approach in a dialectical manner compared to traditional reviews.

## Conclusion

Based on the visual grid analysis established by CiteSpace, we discuss key clusters, established research models, and trends emerging from the reference literature. Through bibliometric analysis, we found that the main areas of knowledge in ISNA research are potential pathogenesis, late stent failure mechanisms and complications, clinical imaging diagnosis of ISNA, and correlation of stent type with the occurrence of ISNA. Pathogenesis and clinical trials are the hot trends of current research in ISNA from the keyword synopsis mapping and co-cited references synopsis mapping. This study uses bibliometrics to explore the research progress in the field of ISNA by analyzing data information from the published literature, which will help researchers to visualize the current status and trends. Compared to traditional reviews, CiteSpace-based bibliometrics has its value but also has the disadvantage of insufficient research depth. However, we still acknowledge and appreciate the contribution of the CiteSpace team and believe that the software will provide more accurate and in-depth analysis of the literature content in the future, as well as provide different perspectives and views for everyone to study related fields.

## Data availability statement

The raw data supporting the conclusions of this article will be made available by the authors, without undue reservation.

## Author contributions

HW and QW conceived the study, carried out the data analysis, interpretation, and manuscript writing, and helped to check the data. JH helped to draft the manuscript. TG helped to collect the data. JH and RZ helped to polish the manuscript. SR and HD conceived the study and made final approval of the manuscript. All authors contributed to the article and approved the submitted version.
