# Supplementary material for: Global research trends in in-stent neoatherosclerosis: A CiteSpace-based visual analysis
Source: Front Cardiovasc Med. 2022 Nov 8;9:1025858. doi: 10.3389/fcvm.2022.1025858 (PMC9679497; doi:10.3389/fcvm.2022.1025858)
Supplement: Supplementary file 1 [file Data_Sheet_1.docx]

**Table S1 |** The top 10 countries/regions with the largest number of articles.

| **Rank** | **Country/regions** | **Count** | **Centrality** | **Number of linked countries** | **Total citations** | **Citations Per Papers** |
| --- | --- | --- | --- | --- | --- | --- |
| 1 | JAPAN | 146 | 0.13 | 10 | 1598 | 10.95 |
| 2 | USA | 136 | 0.29 | 19 | 4474 | 32.9 |
| 3 | CHINA | 61 | 0.13 | 14 | 670 | 10.98 |
| 4 | SOUTH KOREA | 42 | 0.01 | 6 | 1125 | 26.79 |
| 5 | SPAIN | 41 | 0.19 | 14 | 994 | 24.24 |
| 6 | NETHERLANDS | 38 | 0.23 | 20 | 1158 | 30.47 |
| 7 | GERMANY | 31 | 0.05 | 13 | 1345 | 43.39 |
| 8 | ITALY | 29 | 0.18 | 16 | 565 | 19.48 |
| 9 | SWITZERLAND | 22 | 0.07 | 14 | 695 | 31.59 |
| 10 | ENGLAND | 20 | 0.04 | 13 | 554 | 27.70 |

**Table S2 |** The top 10 institutions with the highest number of articles.

| **Rank** | **Institution** | **Count** | **Centrality** | **Number of linked countries** | **Total citations** | **Citations Per Papers** |
| --- | --- | --- | --- | --- | --- | --- |
| 1 | Cardiovasc Res Fdn | 30 | 0.27 | 19 | 818 | 27.27 |
| 2 | Yonsei Univ | 21 | 0.12 | 10 | 455 | 21.67 |
| 3 | CVPath Inst Inc | 19 | 0.14 | 14 | 2023 | 106.47 |
| 4 | Tsuchiura Kyodo Gen Hosp | 18 | 0.03 | 14 | 179 | 9.94 |
| 5 | Kobe Univ | 17 | 0.06 | 5 | 112 | 6.59 |
| 6 | Tech Univ Munich | 17 | 0.04 | 10 | 762 | 44.82 |
| 7 | Columbia Univ | 16 | 0.10 | 15 | 228 | 14.25 |
| 8 | Harbin Med Univ | 16 | 0.10 | 16 | 250 | 15.63 |
| 9 | Harvard Univ | 13 | 0.06 | 15 | 256 | 19.69 |
| 10 | Univ Ulsan | 12 | 0.13 | 10 | 621 | 51.75 |

**
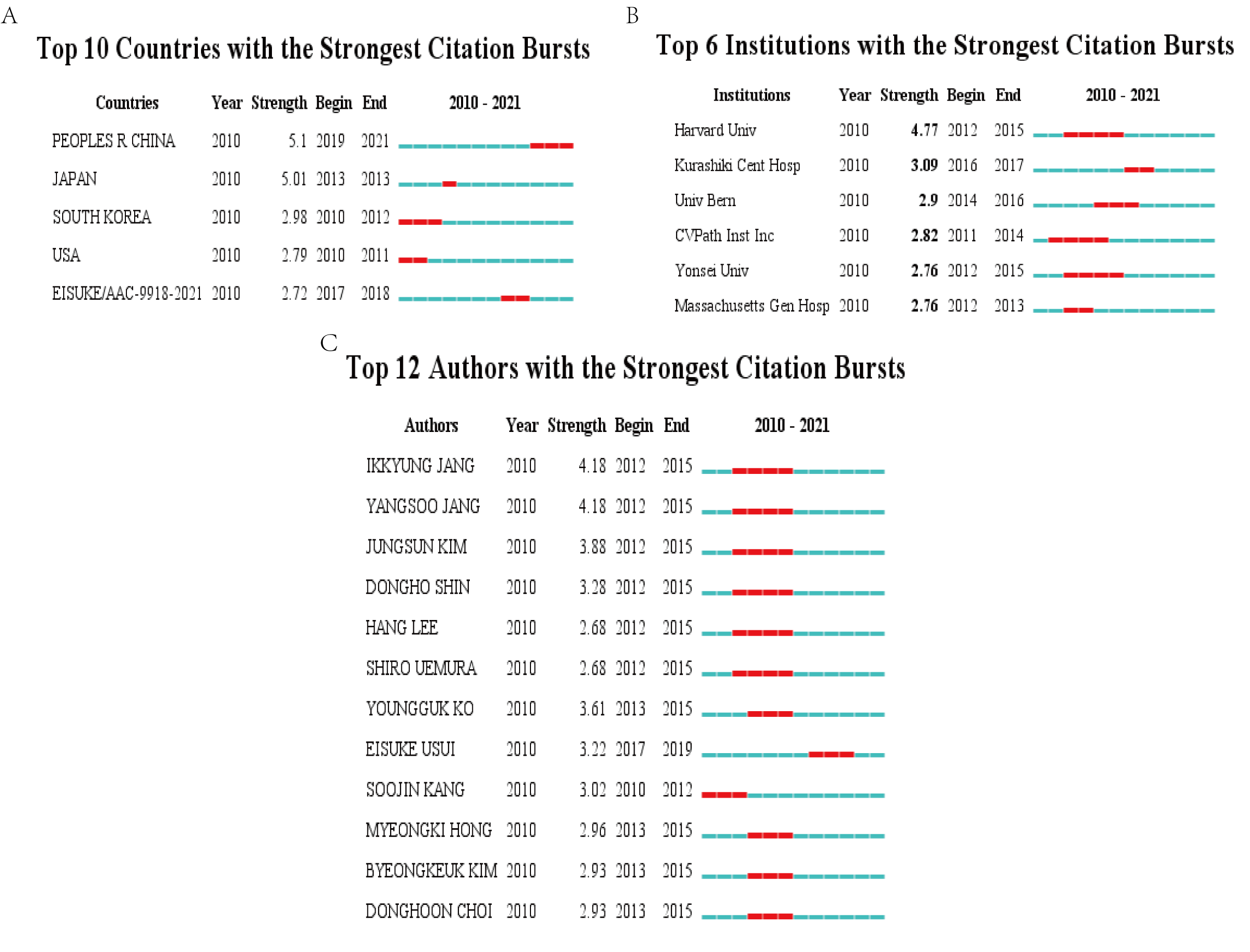
**

**Figure S1 | Burst Mapping. (A)** The top 5 countries/regions with the strongest citation bursts. **(B)** The top 6 institutions with the strongest citation bursts. **(C)** The top 12 authors with the strongest citation bursts.

**Table S3 |** Keyword clustering labels and project members

| **Cluster ID** | **Label (LSI)** | **Cluster member items** |
| --- | --- | --- |
| #0 | neoatherosclerosis | drug-eluting stent; smooth muscle cells; atherosclerosis; polarimetry; coronary artery disease; endothelial cells; restenosis; fibers; endothelial healing |
| #1 | stent thrombosis | optical coherence tomography imaging; acute coronary syndromes; dual antiplatelet therapy; percutaneous coronary intervention; drug-eluting stents; coronary artery calcification; uncovered strut; biodegradable stent; endothelium |
| #2 | stent thrombosis | drug-eluting stents; uncovered strut; percutaneous coronary intervention; coronary artery calcification; dual antiplatelet therapy; coronary artery disease; individual patient data; optical coherence tomography; arterial healing |
| #3 | coronary artery disease | percutaneous coronary intervention; polymer-free; drug-eluting stent; models animal; intravascular ultrasound; stents; renal transplant; myocardial infarction; trial |
| #4 | drug-eluting stent | optical coherence tomography; in-stent restenosis; coronary artery disease; late vascular response; first generation drug-eluting stent; everolimus-eluting stent; individual patient data; intravascular imaging; late stent failure |
| #5 | optical coherence tomography | drug-eluting stent; in-stent restenosis; atherosclerosis; tibial artery; peripheral vascular disease; peroneal artery; infrapopliteal arteries; tibioperoneal artery; below-knee arteries |
| #6 | percutaneous coronary intervention | drug-eluting stent; neoatherosclerosis; bioabsorbable polymer; bioresorbable vascular scaffold; biresorbable polymer; bioresorbable stent; polymer; coronary artery disease; optical coherence tomography |
| #7 | drug-eluting stents | percutaneous coronary intervention; inflammation; biological factors; atherectomy; atherosclerosis; optical coherence tomography; cell coverage; neoatherosclerosis; perilipin |
| #8 | atherosclerosis | lymphocytes; inflammatory disease; macrophages; vascular smooth muscle cell; intermediate calcium-activated potassium channel; neoatherosclerosis; asymmetric dimethylarginine; percutaneous coronary intervention; endothelial cells |

**
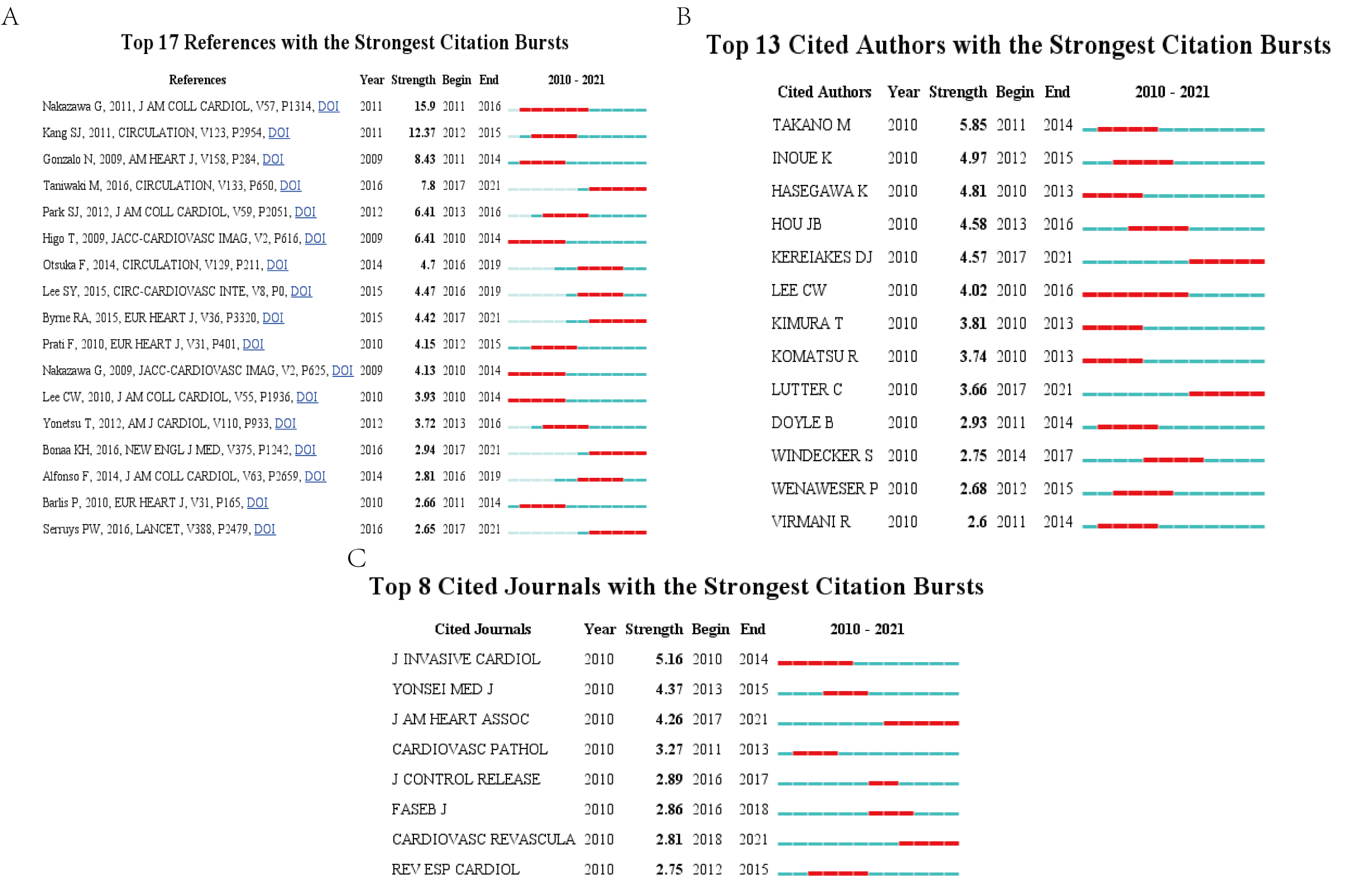
**

**Figure S2 | Burst Mapping.** **(A)** The top 17 references with the strongest citation bursts. **(B)** The top 13 cited authors with the strongest citation bursts. **(C)** The top 8 cited journals with the strongest citation bursts.
